# Correcting Peripherally Inserted Central Catheter Placement by External Manipulation of the Upper Limb Extremity

**DOI:** 10.7759/cureus.66201

**Published:** 2024-08-05

**Authors:** Varun Muddasani, Akshatha P., A. Asha, Harish Sudarsanan, Santosh Kumar Kamalakannan, Kumutha J.

**Affiliations:** 1 Neonatology, Saveetha Medical College and Hospitals, Saveetha Institute of Medical and Technical Sciences, Chennai, IND

**Keywords:** preterm infants, external extremity manipulation, catheter placement, neonatal intensive care units, peripherally inserted central catheters

## Abstract

Peripherally inserted central catheters (PICCs) play a critical role in neonatal intensive care units (NICUs), facilitating treatment in premature and critically ill neonates. However, achieving optimal PICC placement can present challenges, requiring meticulous monitoring and adjustment. Here, we describe the case of a 52-day-old, 1.9 kg preterm infant in the NICU requiring a central venous catheter for antibiotics and antifungals. Despite initial insertion into the basilic vein of the right forearm, imaging revealed the catheter's deviation into the right internal jugular vein. Leveraging the influence of arm position on catheter tip depth, external manipulation of the infant's right arm successfully repositioned the catheter tip into the superior vena cava (SVC). This case highlights the significant impact of arm positioning on PICC placement and underscores the efficacy of external extremity manipulation as a simple, non-invasive technique to adjust catheter position. Such innovative strategies offer promising alternatives to invasive interventions, emphasizing the importance of dynamic monitoring and adjustment techniques in neonatal PICC management.

## Introduction

In neonatal intensive care units (NICUs), peripherally inserted central catheters (PICCs) are vital for treating preterm and severely unwell neonates. PICC line insertion calls for complete aseptic method. Total parenteral nutrition (TPN) and other continuous 24-hour infusions are the main uses for these devices. For extremely low birth weight babies, prolonged central venous access is necessary to provide sufficient nutrition until complete enteral feeding can be established. A small-diameter silicone or polyurethane central venous catheter is inserted percutaneously into a peripheral vein during this procedure. Central venous catheters provide a number of benefits over peripheral cannula, including as better nutrition supply, less phlebitis, and fewer attempts at venous entry per baby. During the first seven days, an umbilical venous line is usually used; if the baby still needs complete parenteral nourishment, a PICC line is then used. It is recommended to place the PICC line before removing the umbilical venous catheter (UVC).

PICC lines may be inserted at a number of locations, the most popular being the long saphenous veins in the lower limbs and the antecubital and basilic veins in the upper limbs. The superior vena cava (SVC) or inferior vena cava (IVC), but obviously outside the cardiac silhouette, is the ideal location for a PICC line's distal tip. The tip of a PICC line placed in an upper limb or scalp vein should be above T4 but still within the SVC. The tip of a PICC line placed in a lower limb should lay on the right side of the spinal column, inside the IVC but below T9.

## Case presentation

**Figure 1:**
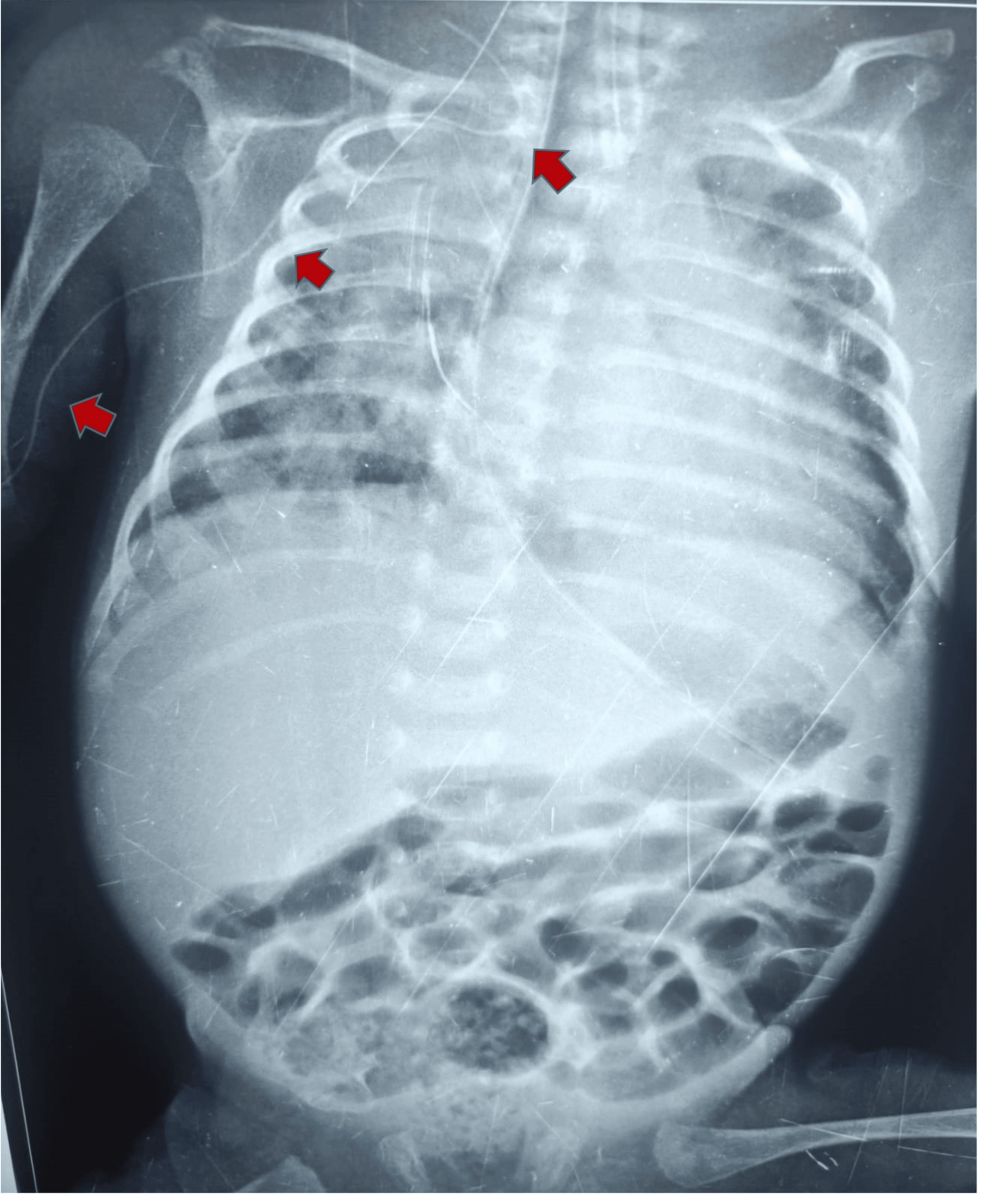
PICC placed from right antecubital vein passing into right internal jugular vein The arrows in the image illustrate the path taken by UVC. PICC: Peripherally inserted central catheter; UVC: Umbilical venous catheter

In the neonatal intensive care unit (NICU) affiliated with Saveetha Medical College and Hospitals, Saveetha Institute of Medical and Technical Sciences, a 52-day-old preterm infant weighing 1.9 kg required a central venous catheter for administering antibiotics and antifungals. Despite initially inserting the catheter into the basilic vein of the right forearm, point-of-care ultrasound and X-ray examinations revealed that the catheter had deviated into the right internal jugular vein as seen in Figure 1. 

**Figure 2:**
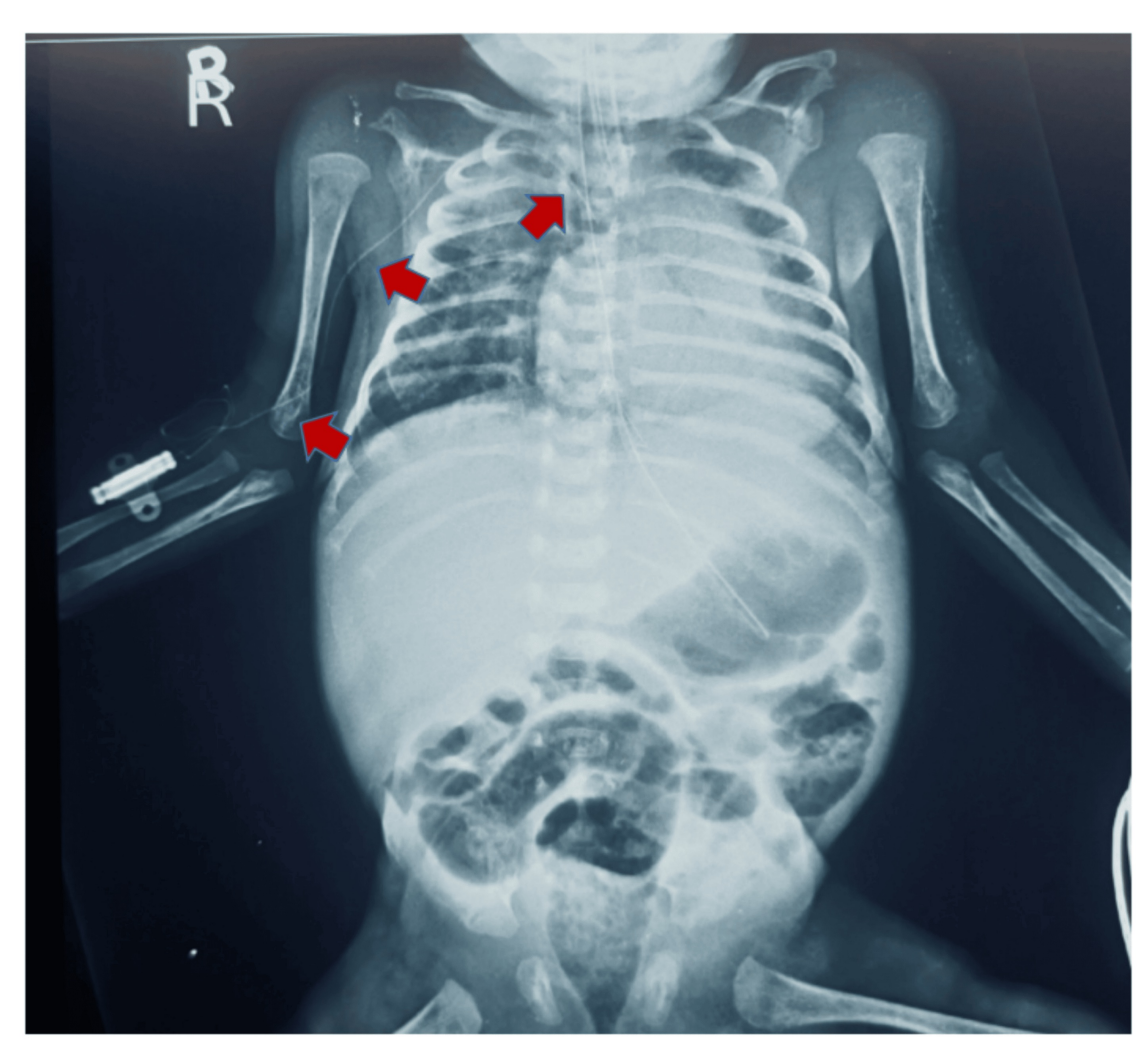
PICC placed correctly in SVC after the external manipulation of arm The arrows in the image illustrate the path taken by UVC and correctly placed in SVC. PICC: Peripherally inserted central catheter; UVC: Umbilical venous catheter; SVC: Superior vena cava

Utilizing the well-known effect of arm positioning on catheter tip depth, medical staff manipulated the infant's right arm to attempt repositioning the catheter tip towards a more peripheral location. The catheter tip moved in a more peripheral direction when the head of the bed was raised and the limb was adjusted, more precisely by abducting at the shoulder joint and straightening the elbow as far as feasible. The catheter tip then moved back into the center by bending the elbow and adducting the shoulder. Following these adjustments, imaging confirmed the catheter's corrected placement in the left SVC (Figure 2). 

## Discussion

PICCs are vital devices in NICUs, which provide accurate drug delivery, including full parenteral feeding and administering of antibiotics and antifungals, to premature and severely sick babies. The following is an extensive explanation of the PICC line insertion process. A face mask, gown, and hand gloves should all be used throughout the sterile procedure of placing a PICC line. Sepsis risk is reduced through broadening the sterile area at the insertion site. In order to prevent catheter-related sepsis, the skin at the insertion site must be effectively cleaned. Aqueous chlorhexidine 0.015% should be administered to the site and let dry for three minutes to ensure that the solution does not pool underneath the baby. After the treatment, any damp or wet linens should be removed immediately. When performing a venepuncture, a transilluminator can help identify the vein and improve accuracy; it can additionally be incorporated into the sterile operation by being covered with a sterile glove.

After inserting the PICC line 2-3 cm longer than expected, it should be pushed back into place. Drawing the blood and flushing it with 0.3 ml of heparin saline (50 IU/5 ml) will guarantee that the line is in a bigger channel and prevent central migration. Avoid applying too much pressure while using the syringe, and always dispose of sharp objects in a plastic container before putting them in a sharps container. To prevent leaks, the PICC catheter must be completely put into the hub, the hub must be tightened, and the blue locking hub must not be removed. The catheter hub should be put on a small roll of sterile gauze, the remaining catheter must be secured to the sterile skin via steristrips, and the insertion site and hub should be covered with an occlusive dressing, avoiding circumferential application.

The following are a few potential complications while using a PICC line. In extensive observational research, out of 2,186 catheters, just one instance of non-lethal pericardial effusion was discovered to be the result of incorrect positioning [1]. 5.3% of catheters developed septicemia, with the tiniest infants having the highest risk of infection. When strict management requirements are followed, such as radiographically verifying the catheter tip location or, in case of uncertainty, using ultrasonic or contrast radiography, the use of subcutaneously implanted central venous catheters is deemed safe. Local edema or infiltration (7.0%), blocked, leaking or bleeding catheters (4.4%), line accidents (1.7%), an irritated insertion site (0.6%), and a malpositioned tip (0.5%) were among the other documented causes for line removal. PICC lines can further raise the risk of thrombosis and air embolism. Common sites for inserting a PICC line in neonates include the arms (basilic, brachial, cephalic, or medial cubital veins, especially the right basilic vein near the elbow), legs (long saphenous veins), and scalp veins. PICCs should not be placed in the hand or wrist [1-6].

**Figure 3:**
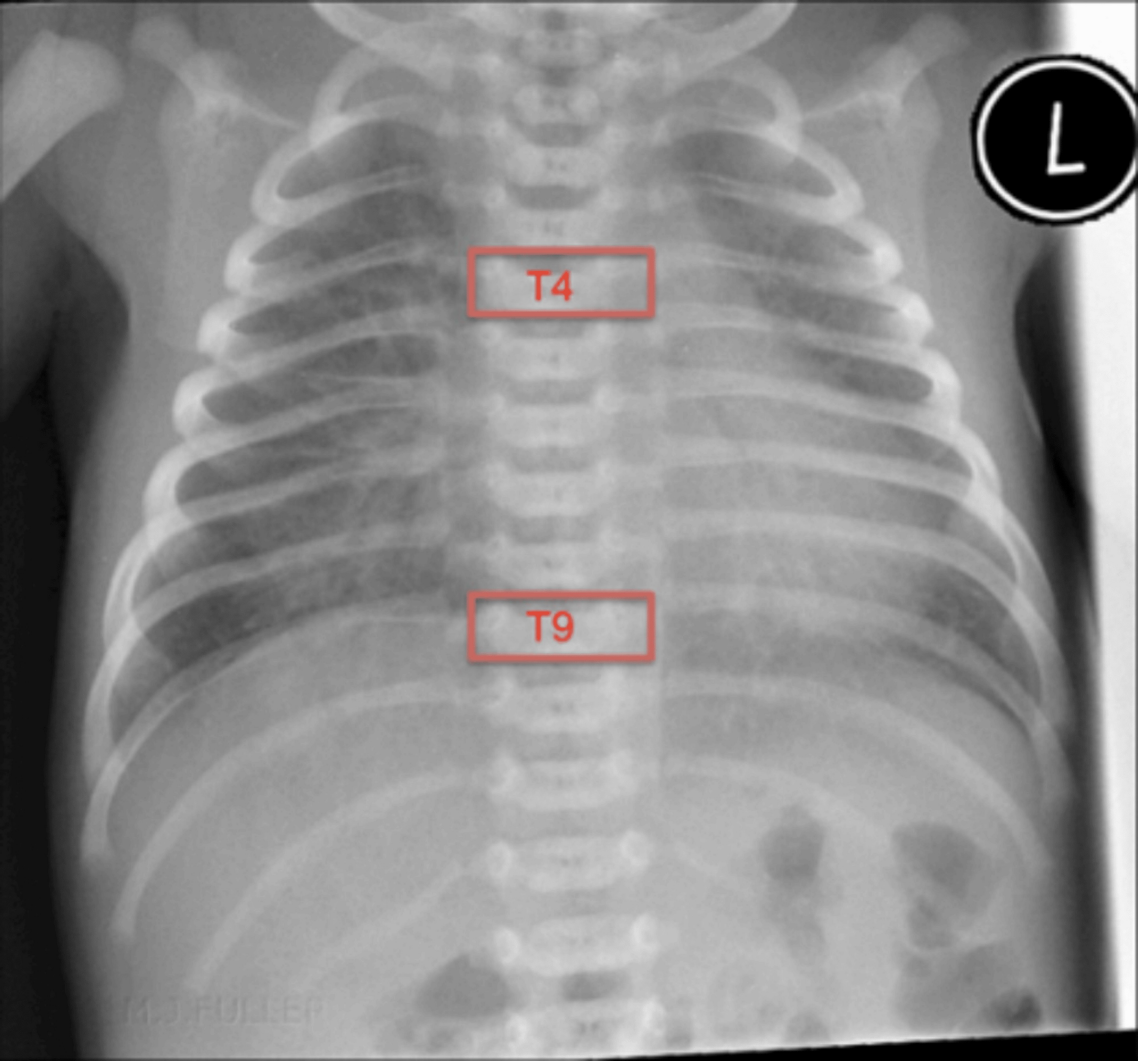
Neonatal CXR with maximal insertion landmarks CXR:

Recent advancements in medical technology have significantly enhanced the management of PICCs in neonates. These advances encompass various aspects, including accurate sizing, innovative vein visualization techniques during insertion, selection of optimal insertion sites, monitoring catheter migration, and confirming PICC tip positions. However, despite these advancements, gaps in understanding and practice persist, indicating areas that necessitate further investigation and research to continually refine PICC management and improve the quality of care for newborns undergoing this procedure [7]. Figure 3 details the maximal insertional landmarks, beyond which PICC placement causes displacement/erroneous placement mandating removal or rectifying the placement of the PICC line.

Neonatologists have enthusiastically embraced these advancements, focusing on meticulous maintenance and removal protocols for PICCs, alongside vigilant monitoring to ensure their proper function and positioning within the infant's vascular system. The dedication to staying updated with current guidelines and integrating innovative practices underscores the commitment to optimizing outcomes for newborns requiring PICC placement [7]. Traditionally, catheter length for insertion into vessels has been determined based on external anatomical landmarks, often leading to the need for post-insertion adjustments due to poor catheter tip positioning, particularly prevalent in newborns (NBs), especially those with lower weights [8-10].

To address this challenge, various formulas have been developed correlating catheter length with NBs' weight and height, depending on the insertion site. For instance, formulas differ for foot, femoral, popliteal, hand, and axillary veins. These formulas have shown promising results in reducing the need for post-insertion catheter adjustments, particularly when inserting into lower limb veins, thereby minimizing the discomfort and stress associated with these procedures for NBs [8]. Recent studies have further refined catheter length determination by establishing formulas that assign constants based on the vein to be punctured and the NB's weight range and birth weight. These formulas have demonstrated high accuracy rates, exceeding 90%, among evaluated NBs. By offering a safe and precise alternative to anatomical landmark-based measurements, these innovative approaches contribute to reducing the incidence of painful and stressful procedures associated with post-insertion catheter adjustments and manipulation of NBs, thus enhancing overall care quality [11].

One particularly noteworthy case study demonstrated the potential of external extremity manipulation as an effective and sterile method to adjust PICC line placement. In a case involving a 2.0 kg early-term infant with congenital heart disease and persistent hypoglycemia, a PICC was initially inserted into the left forearm's basilic vein. However, subsequent imaging revealed its deviation into the left internal jugular vein. Leveraging the influence of arm positioning on catheter tip depth, gentle manipulation of the neonate's left arm facilitated real-time correction of the PICC line's position, ultimately placing it correctly in the left SVC [6]. Another study delved into the intricate relationship between arm movements and PICC tip positioning in neonates. Analyzing radiographs from 60 neonates with PICCs inserted via upper limb veins, the research found that specific arm movements, such as adduction or abduction at the shoulder and flexion or extension at the elbow, significantly influenced catheter displacement. For instance, catheters inserted through the basilic or axillary vein tended to migrate towards the heart with arm adduction. Conversely, those inserted through the cephalic vein moved away from the heart under the same conditions. Notably, the combined movement of shoulder adduction and elbow flexion resulted in the most significant inward movement of basilic vein-inserted catheters, with successful repositioning achieved in nine out of ten cases using arm movements [12].

Arm movements play a pivotal role in determining the position of PICC tips in neonates. To mitigate the risk of catheter migration into the right atrium, it is crucial to identify the insertion vein radiographically and monitor the catheter tip position during movements that induce maximum inward catheter movement for that specific vein [13]. These insights highlight the potential of arm movements as a practical tool for correcting malpositioned catheters and emphasize the importance of meticulous monitoring and adjustment techniques in neonatal PICC management.

## Conclusions

The case of a 52-day-old preterm infant highlights the importance of healthcare providers in ensuring optimal PICC placement. The infant's catheter was initially placed via the basilic vein of the right forearm, but an unintended deviation into the right internal jugular vein required accurate assessment and repositioning. The medical team used non-invasive manoeuvres, such as external extremity manipulation, to redirect the catheter tip towards a more peripheral position. This case highlights the need for continuous imaging guidance and a thorough understanding of neonatal anatomy and physiology in managing central venous catheters.
